# Anti-colorectal cancer effects of seaweed-derived bioactive compounds

**DOI:** 10.3389/fmed.2022.988507

**Published:** 2022-08-19

**Authors:** Yunhua Fu, Dong Xie, Yinghao Zhu, Xinyue Zhang, Hao Yue, Kai Zhu, Zifeng Pi, Yulin Dai

**Affiliations:** ^1^Changchun University of Chinese Medicine, Changchun, China; ^2^Jilin Academy of Agricultural Machinery, Changchun, China

**Keywords:** colorectal cancer, therapeutic compounds, Chlorophyta, Rhodophyta, Phaeophyta

## Abstract

Seaweeds are classified as Chlorophyta, Rhodophyta, and Phaeophyta. They constitute a number of the most significant repositories of new therapeutic compounds for human use. Seaweed has been proven to possess diverse bioactive properties, which include anticancer properties. The present review focuses on colorectal cancer, which is a primary cause of cancer-related mortality in humans. In addition, it discusses various compounds derived from a series of seaweeds that have been shown to eradicate or slow the progression of cancer. Therapeutic compounds extracted from seaweed have shown activity against colorectal cancer. Furthermore, the mechanisms through which these compounds can induce apoptosis *in vitro* and *in vivo* were reviewed. This review emphasizes the potential utility of seaweeds as anticancer agents through the consideration of the capability of compounds present in seaweeds to fight against colorectal cancer.

## Introduction

Colorectal cancer (CRC) is the most common type of cancer throughout the world, accounting for approximately 10% of all new cancer cases and mortality, as projected in GLOBOCAN 2020 (1). The prevalence rates of CRC are increasing among nations with a medium human development index, such as Brazil, Russia, and countries of Latin America (2). The pathology of CRC includes carcinogenesis of the rectum, colon, appendix, and anus (3). Familial and environmental factors contribute to the risk of CRC from two well-defined causes particularly amenable to dietary influence (4). In the clinical situation, chemotherapy is a common treatment modality for CRC (5). Nevertheless, the majority of current chemotherapeutic drugs for the therapy of advanced-stage CRC, for instance cisplatin, have been repeatedly reported to elicit adverse side effects and are comparatively less effective (6). Several lines of scientific evidence, from molecular mechanisms to clinical trials, show that herbal medicines have anti-CRC potential and have been used for therapy and recovery (7).

Seaweeds have been utilized for food and medicinal herbs since ancient times in Asia (8). It has been consumed as a food for over 1,700 years, which can be dated back to Japan in the fourth century and China during sixth century. Particularly, people living long in coastal areas frequently used seaweed as a main dish, side dish, or soup (9). Consumption of seaweed supplies sufficient macro and micronutrients, which are essential to maintaining human health (10). Besides nutritional effects, seaweed has long been adopted as a drug in Traditional East Asian Medicine to alleviate the progression of multiple cancers (11). Seaweeds as large multicellular marine organisms are classified into three major groups based on their pigments and the origin of sulfated polysaccharides: green (Chlorophyta), red (Rhodophyta), and brown (Phaeophyta) (12, 13). They represent a main source of bioactive compounds, yielding primary metabolites essential for natural growth and many secondary metabolites, which include polysaccharides, polyunsaturated fatty acids, phenolics, vitamins, pigments, minerals, terpenes, and phytosterols (14). Due to their various constituents, seaweeds have shown diverse biological activities, including anticancer activity (15).

Seaweeds have long been recognized as a therapeutic option of cancer (16). Accumulating evidence advocates that the anticancer effects of bioactive ingredients extracted from seaweed are produced *via* multiple mechanisms of action, including inhibition of growth, invasion, and metastasis of cancer cells, and through the stimulation of apoptosis in cancer cells (17). Among the East Asian population, people who regularly consumed seaweed reduced their risk of CRC development by half (10). Several researches have suggested that CRC can be effectively treated with marine natural products (18). According to one such report, brown seaweed *Turbinaria decurrens* has the potential as an anti-CRC agent (19). The highly cytotoxic and antiproliferative activities of seaweeds from the Portuguese coast have been proven in a model of Caco-2 CRC cells *in vitro* (20). *Sargassum oligocystom* significantly decreased cell viability in SW742, HT-29, WiDr, and CT-26 CRC cells through activation of the APC gene (21). Although several studies have reported the therapeutic properties of seaweed in CRC, its mechanism of action and active ingredients are still unclear and unclassified. In this review, we summarize the various effects of diverse compounds derived from seaweed on CRC.

## Categorization of anti-CRC compounds isolated from seaweeds

### Polysaccharides

The polysaccharides present in seaweeds are many and diverse (22). They are hydrophilic molecules with high solubility in water and a repeating structure (23). The polysaccharides in seaweed are divided into sulfated (fucoidan, carrageenan, and ulvan) and non-sulfated (agarose and laminarin) (24). Chlorophyta, Phaeophyta, and Rhodophyta contain polysaccharides of varied chemical composition and structure (25).

Previous studies suggested that polysaccharides from seaweed showed strong anti-CRC and preventive activities. They can either directly inhibit cancer cells or affect various phases of carcinogenesis and the progression of tumor through the regulation of the balance between proliferation and programmed cell death and can also be potentially used for cancer prophylaxis (26). Three polysaccharide fractions isolated from *Porphyra haitanensis* exerted inhibitory effects on growth in the HT-29, LoVo, and SW-480 colon cancer cell lines (27). Other active components contained in seaweed that exhibit similar effects against CRC are shown in Table 1. Another study reported on an evaluation of the anti-CRC activity of sulfated glucuronorhamnoxylan polysaccharides from *Capsosiphon fulvescens* (28). Polysaccharides from *Jania rubens* upregulated the gene expression of Bax, caspase 8, and P53 in human colon cancer Caco-2 cells (29). A summary of mechanisms for other active components from seaweed on anti-CRC is shown in Table 2.

**Table 1:** The effects of active components isolated from seaweeds on colorectal cancer.

**Seaweed**	**Division**	**Therapeutic ingredients**	**Cell line**	**IC_50_**	**References**
*Sphaerococcus coronopifolius*	Rhodophyta	Dichloromethane extract	Caco-2	21.3 μg/mL	(20)
*Sargassum oligocystom*	Phaeophyta	Hydroalcoholic extract	CT-26	-	(21)
*Porphyra haitanensis*	Rhodophyta	PHP-F1, PHP-F2 and PHP-F3	HT-29	664.4 μg/mL, 575.1 μg/mL and 578.3 μg/mL	(27)
*Capsosiphon fulvescens*	Chlorophyta	SPS-CF	HT-29	-	(28)
*Jania rubens*	Rhodophyta	*J. rubens* polysaccharide	Caco-2	20 mg/mL	(29)
*Red Seaweeds*	Rhodophyta	AHG	HCT-116	-	(30)
*Ulva lactuca*	Chlorophyta	AgNP	HCT-116	142μM	(31)
*Ulva lactuca*	Chlorophyta	Ulvan polysaccharide	HCT-116	22.65 μg/mL	(32)
*Fucus evanescens*	Phaeophyta	Laminarin	HCT-116	200 μg/mL	(33)
*Kappaphycus alvarezii*	Rhodophyta	κ-carrageenan	HCT-116 HT-29	- 73.87 μg/mL	(34, 35)
*Fucus vesiculosus*	Phaeophyta	Fucoidan	HT-29 HCT-116	200 μg/mL -	(36–38)
*Fucus evanescens*	Phaeophyta	Fucoidan	HCT-116	-	(39)
*Sargassum mcclurei*	Phaeophyta	SmF1, SmF2 and SmF3	DLD-1	-	(40)
*Sargassum glaucescens*	Phaeophyta	SG4	HT-29	272 μg/mL	(41)
*Sargassum cinereum*	Phaeophyta	Fucoidan	Caco-2	250 μg/mL	(42)
*Halimeda opuntia*	Chlorophyta	Carotenoids, chlorophyll a	HT-29	45.23 μg/mL	(43)
*Laminaria japonica*	Phaeophyta	Fucoxanthin	HCT-116	-	(44)
*Sargassum angustifolium*	Phaeophyta	Fucosterol	HT-29	70.41 μg/mL	(45)
*Pterocladiella capillacea*	Rhodophyta	Mertensene	HT-29	56.5 μg/mL	(46)
*Cystoseira usneoides*	Phaeophyta	Meroterpenoids	HT-29	7.8–36.9 μg/mL	(3)

*IC_50_, the half-maximal inhibitory concentration*.

**Table 2:** Properties of active components isolated from seaweed against colorectal cancer.

**Therapeutic ingredients (Seaweed)**	**Cell line**	**Mechanism**	**Cell cycle arrest**	**References**
Hydroalcoholic extract (*Sargassum oligocystom*)	CT-26	Upregulate APC and P53	+	(21)
PHP-F1, PHP-F2 and PHP-F3 (*Porphyra haitanensis*)	HT-29	Induce oxidative stress and apoptosis	G0–G1	(27)
SPS-CF (*Capsosiphon fulvescens*)	HT-29	Upregulate caspase-8,−9,−3 and cleavage of poly (ADP-ribose) polymerase (PARP), induce DNA fragmentation, disrupt MMP	G2/M	(28)
Polysaccharide (*Jania rubens*)	Caco-2	Upregulate Bax, caspase 8 and P53	+	(29)
AHG (Red Seaweeds)	HCT-116	Upregulate Bax, caspase-3,−9 and P53, downregulate Bcl-2 and Bcl-xL	+	(30)
AgNP (*Ulva lactuca*)	HCT-116	Upregulate Bax, P53 and P21, downregulate Bcl-2	+	(31)
Ulvan polysaccharide (*Ulva lactuca*)	HCT-116	Upregulate P53, downregulate Bcl-2	+	(32)
Laminarin (*Fucus evanescens*)	HCT-116	Anti-Proliferation, inhibit MMP-2 and MMP-9 activity	-	(33)
κ-carrageenan (*Kappaphycus alvarezii*)	HCT-116	Induce apoptotic cell death, nuclear fragmentation and apoptosome formation, downregulate XIAP and PARP-1	G1	(34)
Fucoidan (*Fucus vesiculosus*)	HT-29 HCT-116	Increase Bax, caspase-3, PARP-1 and P21, decrease Bcl-2, Cyclin D1 and E, CDK2 and CDK4	G1	(36, 38, 47)
Fucoidan (*Fucus evanescens*)	HCT-116	Decrease TOPK kinase activity, inhibit phosphorylation of TOPK (Thr 9)	-	(39)
SG4 (*Sargassum glaucescens*)	HT-29	Increase cytochrome c release, caspase-9,−3 and DNA fragmentation, disrupt MMP	sub-G1, S, and G2/M	(41)
Fucoidan (*Sargassum cinereum*)	Caco-2	Increase ROS, induce chromatin condensation	-	(42)
Fucoxanthin (*Undaria pinnatidfida*)	Caco-2 DLD-1 HT-29	Upregulate apoptosis, downregulat DNA fragmentation	-	(48)
Fucoxanthin (*Laminaria japonica*)	WiDr HCT-116	Upregulate cell cycle arrest and apoptosis, up-regulation of p21WAF1/Cip1, downregulat proliferation	G0/G1	(44)
Fucoxanthin (Marine algae)	HCT-116 HT29	Upregulate DNA damage	+	(49)
Fucoxanthinol (Brown algae)	DLD-1 HCT-116	Upregulate anoikis and integrin β1, downregulat PPARγ, Akt activation	G1	(50)
Astaxanthin (Marine source)	WiDr	Downregulat proliferation, inhibiting the MYC-mediated downregulation of microRNA-29a-3p and microRNA-200a	-	(51)
ω-3 PUFAs	LOVO	Anti-Proliferation, induce phosphorylation of YAP	-	(52)
EPA	HCT-116	Suppress EGFR and VEGFR activation pathways, downregulate VEGF and HIF1α	-	(53)
DHA	HCT-8 HT-29 HCT-116 SW480	Upregulate TNFα, ERdj5 and caspase-4, downregulate microRNA-21, inhibit RIP1 kinase and AMP-activated protein kinase α	-	(54, 55)
ARA	HT-29	Induce ER stress and apoptosis, inhibit SREBP-1 activity and DNA replication	G1/S	(56, 57)
LA	LOVO CT-26	Upregulate microRNA-494, cytochrome c release, caspase-9,−3 and ROS, downregulate MYCC and PGC1α	S and G2/M	(58, 59)
Fucosterol	HT-29	Anti-Proliferation, upregulate P53, decrease cell viability	+	(60)
Fucosterol (*Sargassum angustifolium*)	HT-29	Induce cytotoxicity	-	(45)
Mertensene (*Pterocladiella capillacea*)	HT-29	Upregulate caspase-3 and cleavage of poly (ADP-ribose) polymerase (PARP), inhibit phosphorylation of P53, Rb, cdc2 and chkp2	G2/M	(46)
Meroterpenoids (*Cystoseira usneoides*)	HT-29	Inhibit phosphorylation of ERK, JKN and AKT	G2/M	(3)
Phloroglucinol	HT-29 HCT-116	Upregulate caspase-3 and caspase-8, inhibited the expression of Ras, Raf, mitogen-activated protein kinase, extracellular-signal regulated kinase phosphorylation, PI3K and Akt	G0/G1	(61)

*+, effects reported; −, no effects reported*.

#### Agarose

Red algae cell walls mainly consist of agarose which is composed of alternative units of D-galactose and 3,6-anhydro-L-galactose (AHG) linked by alternating α-1,3- and β-1,4-glycosidic bonds (62). After being consumed, agarose is digested, fermented, and metabolized by intestinal microbiota in the human large intestine, which makes it unique among red algal polysaccharides (63). Clinical trials have suggested that people in Asia who regularly consume red seaweeds are at a lower risk of CRC, which is relevant to their daily intake of seaweeds (64). It is speculated that this effect may be related to biologically active agarose components enriched from red seaweed. Upregulation of caspase-3, Bax, and caspase-9 expression and downregulation of Bcl-2 and Bcl-xL were observed in HCT-116 cells after AHG treatment (30). Therefore, the growth of human colon cancer HCT-116 cells was effectively suppressed by AHG, indicating that AHG is a potential alternative as an anti-CRC agent.

#### Ulvan

The sulfated polysaccharide known as “ulvan” is extracted from green algae of the ulva species (35). Ulvan mainly consists of cellulose, xyloglucan, and glucuronan with various other types of sugars (65). It is reported to possess diverse physiological and bioactive activities, including anticancer activity (66). Ulvan has been demonstrated to decrease viability in cancer cells while leaving healthy cells unaffected (65). The current study has categorically proven that biogenic silver nanoparticles (AgNP), which were generated *via* an extract of the marine alga *Ulva lactuca*, can induce p53-dependent apoptosis in colon cancer HCT-116 cells (31, 32).

#### Laminarin

Laminarin, sometimes referred to as laminaran, which is an essential biodegradable and non-toxic polysaccharide isolated from the cell wall reservoirs of brown algae, has caught the interest of researchers (67). Laminarins are essentially a group of low-molecular-weight storage β-glucans consisting of (1,3)-β-D-glucan (68). (1,3)-β-D-glucopyranose residues with a few 6-O-branching on the main chain, and also several β-(1,6)-intra-chain links, which are abundant in their structures (69). β-glucans can promote cell apoptosis of colon cancer, and they may be beneficial natural agents for colon cancer treatment and chemoprevention (70). Additionally, certain studies have suggested that the biological activity of laminarin can be strengthened with particular chemical modifications (71). For example, Ji et al. (71) demonstrated that laminarin treated with sulfated provided a stronger antitumor effect compared with unmodified laminarin in human colorectal adenocarcinoma cells. The cell survival rate was significantly decreased after culturing with sulfated laminarin in LoVo cells. Apparently, peculiarities of the polysaccharide structure and sulfation contribute to the anticancer activity of laminarins. Malyarenko et al. (33) found that the antiproliferative activity of laminarins from *Fucus evanescens* was comparable to that of their sulfated derivatives. The anticancer effect of laminarin isolated from *F. evanescens* was stronger than that of its sulfated derivatives in HCT-116 colon carcinoma cells. Ji et al. (72) proved that laminarin increased the intracellular reactive oxygen species (ROS) level, increased intracellular Ca^2+^, decreased intracellular pH, and induced LoVo apoptosis through a mitochondrial pathway. A further study revealed that the expression of procaspase-8 and−3 was downregulated and the activity of caspase-8,−3,−6, and−7 was increased in human colon cancer LoVo cells through the TRAIL/DR pathway after treatment with laminarin (73). Thus, laminarin induces apoptosis in human colon cancer *via* the mitochondrial and DR pathways, indicating that laminarin is a potent anticancer agent.

#### Carrageenan

A set of sulfated polysaccharides generically described as carrageenan is present in red algae, which is the major ingredient of cell walls and interstitial spaces, acting as structural compounds and supplying intercellular adhesion and signaling. The structural units of these natural polysaccharides are a mixture of sulfated linear galactans, which consist of disaccharides of α-(1,4)-linked D-galactopyranose (D) residues or 3,6-anhydrogalactopyranose (DA) and β-(1,3)-linked D-galactopyranose (G) residues (74). According to the concentration, position, and sulfation of 3,6-anhydrogalactose, they are categorized into κ, λ, ι, ν, m, and θ types (75). Polysaccharides with a molecular weight ranging from 500 to 1,000 kDa are present in most of them; however, up to 25% of them may contain polysaccharides at a molecular weight of <100 kDa (76). The significant anticancer and antitumor activities were found in the low-molecular-weight κ- and λ-carrageenan, probably attributed to their antiviral and antioxidant effects as well as the stimulation of immunity against tumors (77). Some studies have reported that the risk of colon cancer appears to be minimized with low-molecular-weight carrageenan, a type of functional food ingredient (34). Carrageenans from *Gigartina pistillata* (78), *Apostichopus japonicus* (79) and *Kappaphycus alvarezii* (80) have an anti-CRC effect on the colon cancer HT-29 cell line. Native carrageenan exerted high cell suppressive activity in colon cancer cells compared with commercial carrageenan. Raman et al. (34) examined the role of the κ-carrageenan-containing soluble dietary fiber fraction of red algae in human colon cancer HCT-116 cells.

However, conflicting studies have suggested that colitis and inflammation may be induced by carrageenan (81). Wei et al. (82) suggested that the existing intestinal inflammation was magnified and TNBS-induced intestinal inflammation was aggravated by κ-carrageenan *via* activating the TLR4-NF-κB and MAPK/ERK1/2 pathways, which indicates it might act as a potential pro-inflammatory factor. In addition, further studies from their group revealed that the LPS-induced inflammation can be synergistically activated by κ-carrageenan through the Bcl10-NF-κB pathway, as illustrated by the aggravation of *Citrobacter freundii* DBS100-induced colitis in mice treated with it (83). Mi et al. (84) investigated the effectiveness of the carrageenan intake form and host intestinal microecology on toxicity in C57BL/6J mice. The severity of colitis in high-fat diet-fed mice could be increased by native carrageenan from drinking water *via* decreasing the abundance of the anti-inflammatory bacterium *Akkermansia muciniphila* and increasing that of harmful bacteria. The inflammatory effect and secretion of proinflammatory cytokines in HT-29 cells can be increased and promoted by using the fermentation supernatants of κ-carrageenan oligosaccharides (85). The inflammatory property of κ-carrageenan oligosaccharides in the context of gut microbiota was evidenced by these results.

#### Fucoidan

Sulfated L-fucose present in the fibrous cell walls and intercellular spaces of brown seaweeds is a major component of fucoidan, which belongs to a large family of marine sulfated polysaccharides (86, 87). Fucoidan is a heparin-like molecule with a simple chemical structure composed of a repeating unit of disaccharides containing α-1,3-linked fucose and α-1,4-linked fucose with branches linked at the C2 positions (86). Generally, L-fucose polymerized with sulfated ester groups is present substantially in fucoidan, while galactose, glucose, mannose, xylose, and glucuronic acid residues are found in only a small proportion (88). Derivatives of fucoidan with a molecular weight varying from 40 to 330 kDa have been examined for their anticancer activities (89). Numerous experiments have shown that tumor cell proliferation and its growth or metastasis can be counteracted by fucoidan *via* eliciting cell apoptosis and suppressing angiogenesis (90). Health can be promoted and gut dysbiosis can be treated by fucoidan, a potential intestinal microbiota modulator. In addition, the effects of induced apoptosis in CRC cells by fucoidan have been evaluated (91).

In HT-29 colon cancer cells, cell viability was reduced by fucoidan in a dose- and time-dependent manner through reducing the expression of CDK2, CDK4, and Cyclin D1 (36). Apoptosis was also mediated by fucoidan *via* inhibition of the IGF-I/IGF-IR/IRS-1/PI3K/AKT (92) and PI3K-Akt-mTOR signaling pathways (37). A further study found that cell cycle arrest at G1-phase was induced by fucoidan *via* the upregulation of p21WAF1 and downregulation of Cyclin D1/CDK4 and Cyclin E/CDK2 expression (47).

In HCT-116 human colon cancer cells, CDK activity was suppressed by fucoidan *via* combining the CDK inhibitor proteins p21 and p27 with the Cyclin/CDK complexes (38). EGF-induced neoplastic cell transformation was significantly inhibited by fucoidan from *F. evanescens via* suppressing the TOPK/ERK1/2/MSK 1 signaling axis (39). In colon cancer cells DLD-1, fucoidan from *Sargassum mcclurei* was observed to be less cytotoxic and inhibited colony formation (40).

Fucoidan strongly regulated the mitochondrial membrane in cancer cells. The evidence found that apoptosis was caused by fucoidan through MMP loss, an increase in cytochrome c release and DNA fragmentation, activated caspase-9 and−3, and an increasing percentage of early and late apoptotic cells in HT-29 cells. Other biological studies indicated that apoptosis was induced by SG4 *via* involvement of the Akt/mTOR/S6 pathway in HT-29 cells (41). Another report showed that fucoidan from *Sargassum cinereum* suppressed the proliferation of Caco-2 cells in a dose-dependent manner, increased ROS production, and augmented mitochondrial membrane permeability (42).

In an animal model, tumor morbidity and average tumor weight were reduced and cellular apoptosis was increased by the treatment of dietary fucoidan in 1,2-dimethylhydrazine (DMH)-induced colorectal carcinogenesis in rats. The expression of β-catenin, C-Myc, Cyclin D1, and Survivin was reduced by treatment with fucoidan, whereas the Hippo pathway was highly activated and the phosphorylation levels of Mst1, Mst2, LATS1, LATS2, and YAP were significantly upregulated (91).

### Pigments

Generally, marine seaweed and animals are rich in pigments, which are widely used in functional food and pharmaceutical industries (93). There are mainly three types of pigments in seaweed involving chlorophylls, carotenoids, and phycobiliproteins (94). The seaweed color was determined by the content and type of pigments (95). For instance, chlorophylls a and b contribute to the green color in green seaweed, whereas allophycocyanin and c-phycoerythrin are responsible for the red color in red seaweed. β-carotene and fucoxanthin (Fx) are commonly observed in brown seaweed (96).

Tumor cells from CRC patients grow uninhibited in the body and enter the blood vessels to spread systemically (97). The apoptosis mechanism is strongly associated with antioxidant properties. Hence, anticancer compounds generally have antioxidant, antiangiogenic, and anti-inflammatory effects to regulate tumor development (98). A recent report showed that the strong antioxidant activity of carotenoids and chlorophyll a from green seaweed *Halimeda opuntia* against HT-29 human colorectal adenocarcinoma was investigated. The finding suggests that natural pigments are potential anticancer ingredients (43).

Carotenoids give seaweed colors from yellow to orangish (99), which of them possess strong activities involving Fx, astaxanthin (AXT), and violaxanthin (100). The evidence showed that carotenoids inhibited the PI3K/Akt apoptosis pathway, eventually integrating the mitochondrial membrane (101).

Fx is one of the most famous lipophilic carotenoids in brown algae, which is responsible for the strong antitumor property. Clinical trials reported that Fx decreased the causing risk of CRC, which has been found to Fx possess potential anti-CRC activity *via* downregulation of tumor-related proteins (102).

In cell experiments, Fx caused a markably decrease of the survival rate in Caco-2, HT-29, DLD-1 (48), and HCT-116 colorectal carcinoma cells (103). It was found that the activation of apoptosis and fragmentation of DNA contributes to the anticancer effect of Fx. Fx showed a significant antiproliferative effect by controlling the level of signaling proteins such as MAPK, NF-κB, and caspase family (99, 104). The disruption of cell cycle arrest causes cell apoptosis. Researchers found that Fx regulated sub-G1 cell cycle arrest in WiDr colon cancer cells (44). Furthermore, Fx stimulated cell cycle arrest at the G0/G1 or G2/M phases and caused programmed cell death (104). It is deduced that Fx induced cell cycle arrest and caused programmed cell death through enhancing intercellular communication between tumor cells.

The derivative compound of Fx was found that exists stronger anticancer activity than Fx. Chemical structural factors significantly influence the antiproliferative properties of Fx. The anti-CRC effect of Fx-degrading compounds was evaluated in Caco-2 cells and its activity may result in partial structures (105). The 13-cis and 13′-cis isomers of Fx showed a markably cancer-preventive effect compared to other derivatives (106). In another study, the antiproliferative effects of 5-fluorouracil (5-Fu) and Fx were determined and compared in HCT-116 and HT-29 cells (49). Fucoxanthinol (FxOH) is the deacetylated type of Fx, which can induce DLD-1 cell apoptosis into anoikis-like changes through the distribution of FAK and integrin β1 expression (50). The mechanism of the anti-CRC effect of FxOH was evaluated *via* regulation of MAPK and STAT apoptosis pathways in HT-29 and HCT-116 cell lines (107), and through inhibiting NF-κB activation in CRC cells (108). According to the previous reports, we found that the anti-CRC activity of FxOH is stronger than Fx, and FxOH induced cancer cells through downregulation of MAPK, STAT, and NF-κB apoptosis pathways. The anticancer effects of Fx and FxOH on six types of CRC cell lines and twenty kinds of tissues from surgically resected clinical CRC specimens were determined as well (109).

In a CRC model animal experiment, a continuous 5-week oral administration of Fx-rich fraction strongly inhibited the number of colorectal adenocarcinomas in DSS-treated male mice (110). Additionally, Fx significantly suppressed colon cancer in azoxymethane-dextrane sodium sulfate (AOM/DSS) carcinogenic model mice (111). In an inflammation-associated CRC mouse model, after a 4-month period of Fx administration, the multiplicity of colorectal adenocarcinoma was strongly decreased *via* upregulated anoikis-like integrin β1^low/−^/cleaved caspase-3^high^ cells in colonic mucosal crypts (112). Additionally, Fx also markedly decreased HSP70 protein in colorectal mucosal crypts for 15-week administration (113). In a 14-week administration in a CRC mouse model, Fx markedly decreased CCR1, pAKT(Ser473), Cyclin D1, and pSmad2 compared with untreated mice (114). Therefore, Fx had chemopreventive potency and therapy ability in the progression of colorectal carcinogenesis in mice.

AXT is mainly contained in seaweed, and showed anti-metastatic activity through inhibiting microRNA-29a-3p and microRNA-200a, thereby downregulating MMP2 and ZEB1 (51). Natural AXT isolated from *Haematococcus pluvialis* showed a significantly anti-CRC effect in a dose-dependent manner in HCT-116 cells by regulating the ratio of Bax/Bcl-2 and upregulating the phosphorylation of p38, JNK, and ERK1/2 (115). AXT induced programmed cell death in DMH-induced rat colon carcinogenesis by modifying NF-κB, COX-2, MMPs-2/9, Akt, and ERK-2 expressions (116). A finding observed that AXT inhibits the progression of colonic premalignant lesions in an obesity-associated colorectal carcinogenesis animal model by attenuating oxidative stress, reducing inflammation and NF-κB activation in the colonic mucosa (117). Hence, AXT is a potential cancer-preventive compound in the therapy of CRC.

### Polyunsaturated fatty acids

Seaweeds are known as low-energy food. Despite their low lipid content, seaweeds contain ω-3 and ω-6 polyunsaturated fatty acids (PUFAs) as a significant portion of their lipids (118). PUFAs are the precursors of eicosanoids and all cell membrane components, which can effectively reduce the risk of cancer (119). Several studies have demonstrated that the colorectal tissue distribution of PUFAs is associated with CRC prognosis (120). There are studies suggesting the potential use of some oxidized metabolites of PUFAs as biomarkers of CRC (121).

#### ω-3 PUFAs

PUFAs in seaweeds contain a substantial amount of ω-3 fatty acids as major components. Eicosapentaenoic acid (EPA) and docosahexaenoic acid (DHA) in seaweed are important ω-3 fatty acids in the marine environment (122). At present, more and more epidemiology and experiments have verified the antitumor activity of ω-3 PUFAs (123). Consumption of diets rich in ω-3 PUFAs not only inhibits the proliferation of CRC cells, but also can be combined with chemotherapy to enhance their sensitivity (124).

PUFAs have pro-apoptotic and growth-inhibitory effects on cancer cells. In LoVo and RKO colon cancer cells, PUFAs can reduce the synthesis of PGE2 and LTB4, inhibit the expression of ALOX5, LTB4, mPGES, COX-2, and PGE2, and increase the expression of LXA4, thereby promoting apoptosis and inhibiting the growth of LoVo and RKO colon cancer cells (125).

In an animal model, dietary supplementation of ω-3 PUFAs increased CRC cell apoptosis and decreased the tumor incidence in AOM/DSS-induced CRC in mice. ω-3 PUFAs treatment activated the hippo pathway, with increased cytoplasmic retention and phosphorylation of YAP (mediated by LATS1 and MST1/2) and the levels of epoxydocosapentaenoic acids (52). Notably, dietary ω-3 PUFAs treatment suppressed the growth of MC38 colorectal tumors. In C57BL/6 mice, ω-3 PUFAs modulate eicosanoid and fatty acid metabolite profiles (126). Huang et al. (127) demonstrated that ω-3 PUFAs reduced tumor incidence in rats by regulation of the DNA methylation process. Together, these results support the notion that ω-3 PUFAs might contribute to the anti-CRC effects of seaweed.

##### EPA

There is evidence of the utility of ω-3 PUFA EPA in the treatment of CRC (128). In a clinical study, patients with familial adenomatous polyposis (FAP) were randomized to receive free fatty acid (FFA). Experimental data proved that EPA 2 g daily in the form of FFA has chemo-preventive efficacy in FAP patients (129). Recently, it has been shown that EPA can prevent FAP-related CRC by acting on several molecular mechanisms (129, 130).

The increased risk of colitis-associated colorectal cancer (CAC) is strongly associated with inflammatory bowel disease, but the effectiveness of dietary EPA-FFA in anti-inflammatory and anticancer activities is unclear. In EPA-FFA-treated AOM-DSS mice, Piazzi et al. (131) found an enrichment of *Lactobacillus species* in the gut microbiota, as well as restored Notch signaling and decreased nuclear β-catenin expression, while tumor cell apoptosis increased. Morin et al. (53) reported that the eicosapentaenoic acid monoglyceride (MAG-EPA) treatment increased HCT-116 cell apoptosis and decreased the tumor of a mouse xenograft model of HCT-116 *via* activating the vascular endothelial growth factor (VEGF) receptor pathway and decreasing the epidermal growth factor receptor (EGFR).

##### DHA

As an ω-3 PUFA, DHA has various biological properties, including anticancer activity (132). The anticancer effect of DHA might be a consequence of its ability to regulate the production of proinflammatory mediators in cancer cells and/or host cells, changing the inflammatory status of the systemic or tumor microenvironment (54, 133). Numerous studies have demonstrated that in CRC cells, the activity of DHA-triggered caspase family members is associated with apoptosis (134). Mechanisms including DHA-induced cellular protein expression explain the antitumor activity of DHA (55).

Fluckiger et al. (54) reported that the TNFα-dependent manner triggers apoptosis in HCT-116, HCT-8, and human CRC cells in DHA-mediated, and cells induced with DHA increased TNFα mRNA content by downregulating microRNA-21 expression, stating that the effect of TNFα on DHA-mediated apoptosis of colon cancer cells. Sarabi et al. (135) demonstrated that DHA could specifically attenuate promoter DNA methylation and VEGF protein levels of microRNA-126 in HCT-116, Caco-2, and CRC cells. Fasano et al. (55) reported that DHA treatment induced apoptosis in HT-29, HCT-116, and SW480 colon cancer cell lines and inhibited their total and surface GRP78 expression, suggesting that pERK1/2 could be the first upstream target of DHA. These studies offer insight into the epigenetic mechanisms by which DHA influences gene expression regulation in CRC cells.

#### ω-6 PUFAs

The ω-6 PUFAs contained in seaweeds include mostly linoleic acid (LA) and arachidonic acid (ARA) (136). Previous studies have suggested that, unlike ω-3 PUFAs, the ω-6 PUFAs, especially ARA, are generally associated with many adverse effects on the human body, including the promotion of multiple cancer types, such as CRC (137, 138). However, there is little *in vitro* evidence to show that ARA exerts anticancer activity in CRC cells. Bae et al. (56) and González-Fernández et al. (57) evidenced that ARA may inhibit DNA replication and G1/S cell cycle transition and induce endoplasmic reticulum stress in HT-29 CRC cells, thereby suppressing cell viability and inducing apoptosis.

Research evidence shows that LA may be involved in both pro- and anticancer activities (137). Lu et al. (139) and Ohmori et al. (140) reported that LA at low concentrations (100-200 μM) reduced caspase-3 activation in CRC cells and promoted tumor cell proliferation. LA at high concentrations (above 300μM) enhanced ROS generation, caused mitochondrial dysfunction and inhibited tumor cell growth (58). A recent report from Ogata et al. (59) suggested that LA induced quiescence by promoting microRNA-494 expression, resulting in the dormancy of CT-26 CRC cells. Therefore, it is speculated that LA can inhibit the growth of CRC cells.

### Phytosterols

Phytosterols are the major nutritional components of seaweed and the most important chemical constituents of algae (141). Phytosterols are the major lipid components of plant cell biofilms. In the marine environment, brown algae are a major source of phytosterols, including brassicosterols with a small amount of plant cholesterol and fucosanols (142). Not only can phytosterols lower cholesterol, they also have strong anticancer activity, and several studies have shown that foods rich in phytosterols may help control the growth of many types of tumors (143).

#### β-Sitosterol

β-Sitosterol, isolated from seaweed, is the most common dietary phytosterol and has a proven potential role in the treatment of CRC (144, 145). Shathviha et al. (146) reported on the evaluation of AgNP synthesized using β-sitosterol and its cytotoxic potential in HT-29 human colon cancer cells. β-Sitosterol-mediated AgNP treatments induced p53 expression and early apoptosis in HT-29 cells. Arul et al. (147) investigated the β-Sitosterol significantly reduces fecal bacteria and colonic bioconverting enzymes in mice with DMH-induced colon cancer, thereby preventing colon cancer development. Amplified activities of colonic biotransformation enzymes are considered hallmarks of colon carcinogenesis. Hence, β-sitosterol is a potential chemopreventive agent in colon carcinogenesis.

#### Fucosterol

The major phytosterol in brown seaweed is fucosterol, which has various biological activities, including anticancer activity (148). A previous study indicated that oxygenated fucosterol inhibited HCT-116 human colon cancer cell growth with higher cytotoxicity than commercial cytotoxic drugs (149). Ramos et al. (60) reported that the combination of fucosterol with 5-Fu can enhance the toxic effect in HT-29 cells. Furthermore, fucosterol is not toxic to normal cells, indicating specificity for cancer cells. The hexane fraction of fucosterol produced by *Sargassum angustifolium* confirmed its cytotoxic activity against HT-29 (45).

### Terpenes

Within the marine environment, terpenes synthesized by algae and secreted to the outside of cells to resist environmental insults are major secondary metabolites from seaweeds (150). It is a chemical compound that contains one or more isoprene units with strong anticancer activity (15). Epidemiological and experimental studies suggest that terpenes may be helpful in curbing the growth of a variety of cancer cells, including colon cells, and provide additional opportunities for cancer therapy (151). Terpenes can modulate pathophysiological processes such as the cell cycle, invasion, migration, proliferation, and apoptosis in different types of tumor cells (152), exhibiting a wide spectrum of antitumor activities (153).

Previous studies have reported that a pentahalogenated monoterpene, halomon, isolated from the red seaweed *Portieria hornemannii*, exhibited strong anticancer activity (154). The halogenated monoterpene mertensene from the red seaweed *Pterocladiella capillacea* (S.G. Gmelin) Santelices & Hommersand inhibited LS174 and HT-29 human colorectal adenocarcinoma cell lines by activating caspase-3 and NF-κB, Akt, and MAPK ERK-1/-2 pathways vitality (46). Terpenes from the brown seaweed *Cystoseira usneoides* have anticancer effects on HT-29 colon cancer cells by reducing the phosphorylation levels of JNK and ERK and inhibiting the ERK/JNK/AKT signaling pathway (3). These reports demonstrate the potential of terpenes as drug candidates for the treatment of colon cancer.

### Phenolics

Phenolic agents are one of the most active compounds in seaweed. Polyphenols with their high molecular weight, such as phlorotannins, specifically exist in brown algae (155). Phlorotannins are composed of a number of phloroglucinol (Ph) monomeric units (156). Those active compounds play a pivotal role in anti-CRC effects, for instance, as apoptotic, anti-metastatic, and antiangiogenic proprieties. They inhibited CRC cell growth directly or indirectly through attenuated inflammatory cytokines and oxidative stress (157). In other reports, the anticancer effects of Ph on insulin-like growth factor-1 receptor signaling in HT-29 human colon cancer cells have been investigated. In addition, Ph inhibited the levels of Ras, mitogen-activated protein kinase, and mTOR (158). The polyphenol-rich agent showed a lower survival rate in CRC cells than the non-polyphenol-rich agent from seaweeds involving *Laminaria japonica, U. lactuca*, and *Porphyra tenera*. Additionally, the polyphenol-rich agent caused G0/G1 cell cycle arrest in HCT-116 cells (159). Phlorofucofuroeckol A (PFF-A) (160) and Ph (161), isolated from brown seaweed, decreased survival rates via activating the apoptosis pathway in CRC cells. Further, Ph decreased the survival rate dose-dependently and induced apoptosis in HT-29 cells, altering Bcl-2 and caspase family proteins (61). The evidence proved that phenolic agents play an important role in understanding the development of colon CRC.

### Vitamins

Seaweed is an important source of various vitamins, among which vitamin C and vitamin D have strong anticancer activity (162). A previous study indicated a potential interplay of vitamin D and immune cells in the tumor microenvironment reduces CRC risk (163). Moreover, some research groups have reported an inverse association between vitamin D3 levels and CRC incidence, and that higher vitamin D3 levels reduce polyp recurrence and improve overall survival in CRC patients (164, 165). Tumor migration and proliferation were inhibited by vitamin D and its analogs in the colon of C57Bl/6 mice (166). Rawson et al. (167) suggested that vitamin D may alter CRC risk by mediating extracellular inhibition. Ferrer-Mayorga et al. (168) indicated that the vitamin D metabolite calcitriol promotes vitamin D receptor expression and inhibits fibroblasts (associated with colon cancer), resulting in anti-CRC effects. There is also a study on vitamin C from Yun et al. (169), who found that cultured CRC cells harboring KRAS or BRAF mutations were selectively killed when exposed to high levels of vitamin C.

### Minerals

The minerals in seaweed are 10–20 times those of land plants and are easily bio-accumulated from seawater (12, 170). The macronutrients (e.g., magnesium, potassium, calcium, and sodium) and microelements (e.g., selenium, iodine, iron, manganese, and zinc) contained in seaweed are important for normal physiological functioning of the body and have potential relevance in cancer treatment (171, 172). Numerous clinical and epidemiological studies indicated that the risk of CRC may be reduced with a higher intake of calcium and magnesium (173). Ali et al. (174) evaluated the antitumor effect of selenium against DMH-induced CRC in BALB/C mice and its effect on apoptosis and angiogenesis. The group treated with DMH plus selenium exhibited significantly lower expression of cloned caudal-type homeobox gene-2 and VEGF but a higher caspase-3 expression level than the DMH-treated group.

## Conclusions

Many studies have demonstrated the effect and mechanism of red, green, and brown seaweeds in CRC-prevention and therapy. Various therapeutic compounds from seaweed involving large molecular polysaccharides and small molecular pigments, fatty acids, phytosterols, terpenes, phenolics, vitamins and minerals induce programmed cell death *via* various signaling pathways. Such treatments can alter the protein expression of Bax, caspases, Bcl-2, MAPK, NF-κB, VEGF, DNA methylation, and CDK inhibitor and induce changes in the cell cycle and the CRC cellular functions of adhesion, migration, and invasion. Important anti-CRC agents such as polysaccharides and fatty acids existed for their antiproliferative and anti-metastatic effects *in vivo*. Furthermore, terpenes and pigments inhibited CRC cell survival rate and induced programmed cell death *via* regulation of the Akt signaling pathway.

A few molecular alterations in human CRC cells were correspondingly observed in CRC animal models treated with seaweed. Consecutive studies *in vitro* will be important as the basis for clarifying the molecular mechanisms underlying cancer prevention in humans with CRC and CRC animal models. CRC animal models, AOM-DSS, BALB/C, and C57BL/6 mice, have been the vehicles for many discoveries concerning the anti-CRC effects of seaweed. The oxidation, inflammation and gut microbiota, which are significant factors associated with colorectal carcinogenesis, have been reported to be prime targets of various therapeutic compounds from seaweed. In addition, the administration of Fx induced anoikis in CRC animal models. However, the detailed molecular mechanisms underlying the cancer chemopreventive effect in animals remain poorly understood.

Finally, this review highlights the importance of seaweed as a potential agent candidate for preventing CRC. However, the underlying mechanisms remain elusive. Further clinical investigations are needed to assess the anticancer effect of seaweed in humans.

## Author contributions

YD and ZP conceptualized the idea and scope of the review. YZ, XZ, HY, and KZ both performed a literature review. YF and DX wrote the manuscript and created the figures. All authors critically revised and edited the manuscript.

## Funding

This research was funded by the Scientific and Technological Development Program of Jilin Province of China, grant numbers (20210401065YY and 20210204046YY).

## Conflict of interest

The authors declare that the research was conducted in the absence of any commercial or financial relationships that could be construed as a potential conflict of interest.

## Publisher's note

All claims expressed in this article are solely those of the authors and do not necessarily represent those of their affiliated organizations, or those of the publisher, the editors and the reviewers. Any product that may be evaluated in this article, or claim that may be made by its manufacturer, is not guaranteed or endorsed by the publisher.

## References

[1] Sung H Ferlay J Siegel RL Laversanne M Soerjomataram I Jemal A. Global cancer statistics 2020: globocan estimates of incidence and mortality worldwide for 36 cancers in 185 countries. CA Cancer J Clin. (2021) 71:209–49. 10.3322/caac.21660 33538338

[2] Rawla P Sunkara T Barsouk A. Epidemiology of colorectal cancer: incidence, mortality, survival, and risk factors. Prz Gastroenterol. (2019) 14:89–103. 10.5114/pg.2018.81072 31616522 PMC6791134

[3] Zbakh H Zubia E Reyes CL Calderon-Montano JM Motilva V. Anticancer activities of meroterpenoids isolated from the brown alga cystoseira usneoides against the human colon cancer cells Ht-29. Foods. (2020) 9:300. 10.3390/foods9030300 32155797 PMC7143549

[4] Davis CD Milner JA. Molecular targets for nutritional preemption of cancer. Curr Cancer Drug Targets. (2007) 7:410–5. 10.2174/156800907781386560 17691899

[5] Huang G Liu Z He L Luk KH Cheung ST Wong KH. Autophagy is an important action mode for functionalized selenium nanoparticles to exhibit anti-colorectal cancer activity. Biomater Sci. (2018) 6:2508–17. 10.1039/C8BM00670A 30091749

[6] Dasari S Tchounwou PB. Cisplatin in cancer therapy: molecular mechanisms of action. Eur J Pharmacol. (2014) 740:364–78. 10.1016/j.ejphar.2014.07.025 25058905 PMC4146684

[7] Feng Y Wang N Zhu M Feng Y Li H Tsao S. Recent progress on anticancer candidates in patents of herbal medicinal products. Recent Pat Food Nutr Agric. (2011) 3:30–48. 10.2174/2212798411103010030 21114469

[8] Desideri D Cantaluppi C Ceccotto F Meli MA Roselli C Feduzi L. Essential and toxic elements in seaweeds for human consumption. J Toxicol Environ Health A. (2016) 79:112–22. 10.1080/15287394.2015.1113598 26817952

[9] Kilinç B Cirik S Turan G Tekogul H Koru E. Seaweeds for food and industrial applications. In: Muzzalupo, editor. Food Industry IntechOpen. Izmir (2013). p. 735–48. 10.5772/53172

[10] Cotas J Pacheco D Gonçalves AMM Silva P Carvalho LG Pereira L. Seaweeds' nutraceutical and biomedical potential in cancer therapy: a concise review. J Cancer Metast Treat. (2021) 7:13. 10.20517/2394-4722.2020.134

[11] Brown EM Allsopp PJ Magee PJ Gill CI Nitecki S Strain CR. Seaweed and Human Health. Nutr Rev. (2014) 72:205–16. 10.1111/nure.12091 24697280

[12] Makkar HP Tran G Heuzé V Giger-Reverdin S Lessire M Lebas F. Seaweeds for livestock diets: a review. Anim Feed Sci Technol. (2016) 212:1–17. 10.1016/j.anifeedsci.2015.09.018

[13] Arokiarajan MS Thirunavukkarasu R Joseph J Ekaterina O Aruni W. Advance research in biomedical applications on marine sulfated polysaccharide. Int J Biol Macromol. (2022) 194:870–81. 10.1016/j.ijbiomac.2021.11.142 34843816

[14] Murphy C Hotchkiss S Worthington J McKeown SR. The potential of seaweed as a source of drugs for use in cancer chemotherapy. J Appl Phycol. (2014) 26:2211–64. 10.1007/s10811-014-0245-2

[15] Gutierrez-Rodriguez AG Juarez-Portilla C Olivares-Banuelos T Zepeda RC. Anticancer activity of seaweeds. Drug Discov Today. (2018) 23:434–47. 10.1016/j.drudis.2017.10.019 29107095

[16] Namvar F Baharara J Mahdi AA. Antioxidant and anticancer activities of selected persian gulf algae. Indian J Clin Biochem. (2014) 29:13–20. 10.1007/s12291-013-0313-4 24478544 PMC3903930

[17] Farooqi AA Butt G Razzaq Z. Algae extracts and methyl jasmonate anti-cancer activities in prostate cancer: choreographers of ‘the dance macabre'. Cancer Cell Int. (2012) 12:1–6. 10.1186/1475-2867-12-50 23181808 PMC3575221

[18] Ryu MJ Kim AD Kang KA Chung HS Kim HS Suh IS. The green algae ulva fasciata delile extract induces apoptotic cell death in human colon cancer cells. In Vitro Cell Dev Biol Anim. (2013) 49:74–81. 10.1007/s11626-012-9547-3 23299316

[19] Zakaria AD Basah K Bahtiar A Deviyani Z Basah K Bahtiar A. Cytotoxic activity of extract and active fraction of turbinaria decurrens bory on colon cancer cell Line Hct-116. Int J Morphol. (2018) 36:979–83. 10.4067/S0717-95022018000300979

[20] Alves C Pinteus S Rodrigues A Horta A Pedrosa R. Algae from portuguese coast presented high cytotoxicity and antiproliferative effects on an in vitro model of human colorectal cancer. Pharmacognosy Res. (2018) 10:24-30. 10.4103/pr.pr_151_16 29568183 PMC5855369

[21] Ghazy E Fadil AG Hussen BM Al-Marzoqi AH. Anticancer Effect of Sargassum Oligocystom Hydroalcoholic Extract against Sw742, Ht-29, Widr and Ct-26 Colorectal Cancer Cell Lines and Expression of P53 and Apc Genes. (2021). 10.21203/rs.3.rs-906543/v1 35000070

[22] Sakthivel R Devi KP. Antioxidant, anti-inflammatory and anticancer potential of natural bioactive compounds from seaweeds. Stud Nat Prod Chem. (2019) 63:113–60. 10.1016/B978-0-12-817901-7.00005-831683523

[23] Pereira L. Seaweeds as source of bioactive substances and skin care therapy—cosmeceuticals, algotheraphy, and thalassotherapy. Cosmetics. (2018) 5:68. 10.3390/cosmetics5040068

[24] Hentati F Tounsi L Djomdi D Pierre G Delattre C Ursu AV. Bioactive polysaccharides from seaweeds. Molecules. (2020) 25:3152. 10.3390/molecules25143152 32660153 PMC7397078

[25] Wang Z Xie J Shen M Nie S Xie M. Sulfated modification of polysaccharides: synthesis, characterization and bioactivities. Trends Food Sci Technol. (2018) 74:147–57. 10.1016/j.tifs.2018.02.010

[26] Fedorov SN Ermakova SP Zvyagintseva TN Stonik VA. Anticancer and cancer preventive properties of marine polysaccharides: some results and prospects. Mar Drugs. (2013) 11:4876–901. 10.3390/md11124876 24317475 PMC3877892

[27] Yao W-Z Veeraperumal S Qiu H-M Chen X-Q Cheong K-L. Anti-cancer effects of porphyra haitanensis polysaccharides on human colon cancer cells via cell cycle arrest and apoptosis without causing adverse effects in vitro. 3 Biotech. (2020) 10:1–11. 10.1007/s13205-020-02379-y 32832336 PMC7419411

[28] Choi JW Lee J Kim SC You S Lee CW Shin J. Glucuronorhamnoxylan from capsosiphon fulvescens inhibits the growth of Ht-29 human colon cancer cells in vitro and in vivo via induction of apoptotic cell death. Int J Biol Macromol. (2019) 124:1060–8. 10.1016/j.ijbiomac.2018.12.001 30521889

[29] Gheda S El-Sheekh M Abou-Zeid A. In vitro anticancer activity of polysaccharide extracted from red alga jania rubens against breast and colon cancer cell lines. Asian Pac J Trop Med. (2018) 11:583. 10.4103/1995-7645.244523

[30] Yun EJ Yu S Kim YA Liu JJ Kang NJ Jin YS. In vitro prebiotic and anti-colon cancer activities of agar-derived sugars from red seaweeds. Mar Drugs. (2021) 19:213. 10.3390/md19040213 33921308 PMC8070132

[31] Acharya D Satapathy S Yadav KK Somu P Mishra G. Systemic evaluation of mechanism of cytotoxicity in human colon cancer Hct-116 cells of silver nanoparticles synthesized using marine algae ulva lactuca extract. J Inorg Organomet Polym Mater. (2022) 32:596–605. 10.1007/s10904-021-02133-8

[32] Ahmed O Ahmed R. Anti-proliferative and apoptotic efficacies of ulvan polysaccharides against different types of carcinoma cells. vitro and in vivo. J Cancer Sci Ther. (2014) 6:202–8. 10.4172/1948-5956.1000272

[33] Malyarenko OS Usoltseva RV Shevchenko NM Isakov VV Zvyagintseva TN Ermakova SP. In vitro anticancer activity of the laminarans from far eastern brown seaweeds and their sulfated derivatives. J Appl Phycol. (2017) 29:543–53. 10.1007/s10811-016-0915-3

[34] Raman M Doble M. κ-Carrageenan from marine red algae, kappaphycus alvarezii–a functional food to prevent colon carcinogenesis. J Funct Foods. (2015) 15:354–64. 10.1016/j.jff.2015.03.037

[35] Morelli A Puppi D Chiellini F. Perspectives on Biomedical Applications of Ulvan. Seaweed Polysaccharides. Elsevier (2017). p. 305–30. 10.1016/B978-0-12-809816-5.00016-5

[36] Yun CW Yun S Lee JH Han Y-S Yoon YM An D. Silencing prion protein in Ht29 human colorectal cancer cells enhances anticancer response to fucoidan. Anticancer Res. (2016) 36:4449–58. 10.21873/anticanres.10989 27630281

[37] Han YS Lee JH Lee SH. Fucoidan inhibits the migration and proliferation of Ht-29 human colon cancer cells via the phosphoinositide-3 Kinase/Akt/Mechanistic target of rapamycin pathways. Mol Med Rep. (2015) 12:3446–52. 10.3892/mmr.2015.3804 25998232 PMC4526071

[38] Park HY Park S-H Jeong J-W Yoon D Han MH Lee D-S. Induction of P53-independent apoptosis and G1 cell cycle arrest by fucoidan in Hct116 human colorectal carcinoma cells. Mar Drugs. (2017) 15:154. 10.3390/md15060154 28555064 PMC5484104

[39] Vishchuk OS Sun H Wang Z Ermakova SP Xiao J Lu T. Pdz-binding kinase/T-Lak cell-originated protein kinase is a target of the fucoidan from brown alga fucus evanescens in the prevention of Egf-induced neoplastic cell transformation and colon cancer growth. Oncotarget. (2016) 7:18763. 10.18632/oncotarget.7708 26936995 PMC4951327

[40] Thinh PD Menshova RV Ermakova SP Anastyuk SD Ly BM Zvyagintseva TN. Structural characteristics and anticancer activity of fucoidan from the brown alga sargassum mcclurei. Mar Drugs. (2013) 11:1456–76. 10.3390/md11051456 23648551 PMC3707154

[41] Shiao W-C Kuo C-H Tsai Y-H Hsieh S-L Kuan A-W Hong Y-H. In vitro evaluation of anti-colon cancer potential of crude extracts of fucoidan obtained from sargassum glaucescens pretreated by compressional-puffing. Applied Sciences. (2020) 10:3058. 10.3390/app10093058

[42] Narayani SS Saravanan S Ravindran J Ramasamy M Chitra J. In vitro anticancer activity of fucoidan extracted from sargassum cinereum against Caco-2 cells. Int J Biol Macromol. (2019) 138:618–28. 10.1016/j.ijbiomac.2019.07.127 31344415

[43] Nazarudin M Yasin I Mazli N Saadi A Azizee M Nooraini M. Preliminary screening of antioxidant and cytotoxic potential of green seaweed, halimeda opuntia (linnaeus) lamouroux. Saudi J Biol Sci. (2022) 29:2698–705. 10.1016/j.sjbs.2021.12.066 35531161 PMC9073034

[44] Das SK Hashimoto T Shimizu K Yoshida T Sakai T Sowa Y. Fucoxanthin induces cell cycle arrest at G0/G1 phase in human colon carcinoma cells through up-regulation of P21waf1/Cip1. Biochimica et Biophysica Acta. (2005) 1726:328–35. 10.1016/j.bbagen.2005.09.007 16236452

[45] Khanavi M Gheidarloo R Sadati N Ardekani MRS Nabavi SMB Tavajohi S. Cytotoxicity of fucosterol containing fraction of marine algae against breast and colon carcinoma cell line. Pharmacogn Mag. (2012) 8:60. 10.4103/0973-1296.93327 22438665 PMC3307205

[46] Tarhouni-Jabberi S Zakraoui O Ioannou E Riahi-Chebbi I Haoues M Roussis V. Mertensene, a halogenated monoterpene, induces G2/M cell cycle arrest and caspase dependent apoptosis of human colon adenocarcinoma Ht29 cell line through the modulation of Erk-1/-2, Akt and Nf-κb signaling. Mar Drugs. (2017) 15:221. 10.3390/md15070221 28726723 PMC5532663

[47] Han Y-s Lee JH Lee SH. Antitumor effects of fucoidan on human colon cancer cells via activation of Akt signaling. Biomol Ther. (2015) 23:225. 10.4062/biomolther.2014.136 25995820 PMC4428714

[48] Hosokawa M Kudo M Maeda H Kohno H Tanaka T Miyashita K. Fucoxanthin induces apoptosis and enhances the antiproliferative effect of the Pparγ ligand, troglitazone, on colon cancer cells. Biochimica et Biophysica Acta. (2004) 1675:113–9. 10.1016/j.bbagen.2004.08.012 15535974

[49] Lopes-Costa E Abreu M Gargiulo D Rocha E Ramos AA. Anticancer effects of seaweed compounds fucoxanthin and phloroglucinol, alone and in combination with 5-fluorouracil in colon cells. J Toxicol Environ Health A. (2017) 80:776–87. 10.1080/15287394.2017.1357297 28850007

[50] Terasaki M Maeda H Miyashita K Mutoh M. Induction of anoikis in human colorectal cancer cells by fucoxanthinol. Nutr Cancer. (2017) 69:1043–52. 10.1080/01635581.2017.1339814 28990814

[51] Kim H-Y Kim Y-M Hong S. Astaxanthin suppresses the metastasis of colon cancer by inhibiting the Myc-mediated downregulation of microrna-29a-3p and microrna-200a. Sci Rep. (2019) 9:1–10. 10.1038/s41598-019-45924-3 31263239 PMC6603017

[52] Zhang K Hu Z Qi H Shi Z Chang Y Yao Q. G-protein-coupled receptors mediate Ω-3 pufas-inhibited colorectal cancer by activating the hippo pathway. Oncotarget. (2016) 7:58315. 10.18632/oncotarget.11089 27506947 PMC5295433

[53] Morin C Rodríguez E Blier PU Fortin S. Potential application of eicosapentaenoic acid monoacylglyceride in the management of colorectal cancer. Mar Drugs. (2017) 15:283. 10.3390/md15090283 28869531 PMC5618422

[54] Fluckiger A Dumont A Derangere V Rebe C De Rosny C Causse S. Inhibition of colon cancer growth by docosahexaenoic acid involves autocrine production of Tnfα. Oncogene. (2016) 35:4611–22. 10.1038/onc.2015.523 26853468

[55] Fasano E Serini S Piccioni E Toesca A Monego G Cittadini AR. Dha induces apoptosis by altering the expression and cellular location of Grp78 in colon cancer cell lines. Biochimica et Biophysica Acta. (2012) 1822:1762–72. 10.1016/j.bbadis.2012.08.003 22898250

[56] Bae S Kim M-K Kim HS Moon Y-A. Arachidonic acid induces er stress and apoptosis in Ht-29 human colon cancer cells. Animal Cells Syst. (2020) 24:260–6. 10.1080/19768354.2020.1813805 33209199 PMC7646553

[57] González-Fernández MJ Fabrikov D Ramos-Bueno RP Guil-Guerrero JL Ortea I. Swath differential abundance proteomics and cellular assays show in vitro anticancer activity of arachidonic acid-and docosahexaenoic acid-based monoacylglycerols in Ht-29 colorectal cancer cells. Nutrients. (2019) 11:2984. 10.3390/nu11122984 31817645 PMC6950369

[58] Lu X Yu H Ma Q Shen S Das UN. Linoleic acid suppresses colorectal cancer cell growth by inducing oxidant stress and mitochondrial dysfunction. Lipids Health Dis. (2010) 9:1–11. 10.1186/1476-511X-9-106 20868498 PMC2954911

[59] Ogata R Mori S Kishi S Sasaki R Iwata N Ohmori H. Linoleic acid upregulates microrna-494 to induce quiescence in colorectal cancer. Int J Mol Sci. (2021) 23:225. 10.3390/ijms23010225 35008652 PMC8745195

[60] Ramos AA Almeida T Lima B Rocha E. Cytotoxic activity of the seaweed compound fucosterol, alone and in combination with 5-fluorouracil, in colon cells using 2d and 3d culturing. J Toxicol Environ Health A. (2019) 82:537–49. 10.1080/15287394.2019.1634378 31258008

[61] Kang M-H Kim I-H Nam T-J. Phloroglucinol induces apoptosis via apoptotic signaling pathways in Ht-29 colon cancer cells. Oncol Rep. (2014) 32:1341–6. 10.3892/or.2014.3355 25070748 PMC4148371

[62] Knutsen S Myslabodski D Larsen B Usov A. A Modified System of Nomenclature for Red Algal Galactans. (1994). 10.1515/botm.1994.37.2.163

[63] Shang Q Jiang H Cai C Hao J Li G Yu G. Gut microbiota fermentation of marine polysaccharides and its effects on intestinal ecology: an overview. Carbohydr Polym. (2018) 179:173–85. 10.1016/j.carbpol.2017.09.059 29111040

[64] Kim J Lee J Oh JH Chang HJ Sohn DK Shin A. Associations among dietary seaweed intake, C-Myc Rs6983267 polymorphism, and risk of colorectal cancer in a korean population: a case–control study. Eur J Nutr. (2020) 59:1963–74. 10.1007/s00394-019-02046-w 31300834

[65] Cindana Mo'o FR Wilar G Devkota HP Wathoni N. Ulvan, a polysaccharide from macroalga ulva sp.: a review of chemistry, biological activities and potential for food and biomedical applications. Appl Sci. (2020) 10:5488. 10.3390/app10165488

[66] Thanh TTT Quach TMT Nguyen TN Luong DV Bui ML Van Tran TT. Structure and cytotoxic activity of ulvan extracted from green seaweed ulva lactuca. Int J Biol Macromol. (2016) 93:695–702. 10.1016/j.ijbiomac.2016.09.040 27637450

[67] Zargarzadeh M Amaral AJ Custódio CA Mano JF. Biomedical applications of laminarin. Carbohydr Polym. (2020) 232:115774. 10.1016/j.carbpol.2019.115774 31952585

[68] Rioux L-E Turgeon SL Beaulieu M. Characterization of polysaccharides extracted from brown seaweeds. Carbohydr Polym. (2007) 69:530–7. 10.1016/j.carbpol.2007.01.009

[69] Kadam SU Tiwari BK O'Donnell CP. Extraction, structure and biofunctional activities of laminarin from brown algae. Int J Food Sci Technol. (2015) 50:24–31. 10.1111/ijfs.12692

[70] Kim M-J Hong S-Y Kim S-K Cheong C Park H-J Chun H-K. β-Glucan enhanced apoptosis in human colon cancer cells Snu-C4. Nutr Res Pract. (2009) 3:180–4. 10.4162/nrp.2009.3.3.180 20090882 PMC2808716

[71] Ji C-F Ji Y-B Meng D-Y. Sulfated modification and anti-tumor activity of laminarin. Exp Ther Med. (2013) 6:1259–64. 10.3892/etm.2013.1277 24223655 PMC3820847

[72] Ji YB Ji CF Zhang H. Laminarin induces apoptosis of human colon cancer lovo cells through a mitochondrial pathway. Molecules. (2012) 17:9947–60. 10.3390/molecules17089947 22907156 PMC6268208

[73] Ji CF Ji YB. Laminarin-induced apoptosis in human colon cancer lovo cells. Oncol Lett. (2014) 7:1728–32. 10.3892/ol.2014.1952 24765209 PMC3997718

[74] Khotimchenko M Tiasto V Kalitnik A Begun M Khotimchenko R Leonteva E. Antitumor potential of carrageenans from marine red algae. Carbohydr Polym. (2020) 246:116568. 10.1016/j.carbpol.2020.116568 32747241

[75] Necas J Bartosikova L. Carrageenan: a review. Vet Med. (2013) 58:187–205. 10.17221/6758-VETMED

[76] Lascombes C Agoda-Tandjawa G Boulenguer P Le Garnec C Gilles M Mauduit S. Starch-carrageenan interactions in aqueous media: role of each polysaccharide chemical and macromolecular characteristics. Food Hydrocoll. (2017) 66:176–89. 10.1016/j.foodhyd.2016.11.025

[77] Zhou G Sun Y Xin H Zhang Y Li Z Xu Z. In vivo antitumor and immunomodulation activities of different molecular weight lambda-carrageenans from chondrus ocellatus. Pharmacol Res. (2004) 50:47–53. 10.1016/j.phrs.2003.12.002 15082028

[78] Cotas J Marques V Afonso MB Rodrigues CM Pereira L. Carrageenans Extracted from Gigartina Pistillata Demonstrate Potential against Colorectal Cancer Stem Cell-Enriched Tumorspheres. In: Conference: Conferência APAA 2019 - Associação Portuguesa de Algologia Aplicada (APAA). Coimbra; Lisbon (2019). 10.13140/RG.2.2.17470.02889

[79] Yu-lin D Dong-yue Z Yun-Fei J Fei Z Hao Y You-Jin J. 6-Bromohypaphorine isolated from red sea cucumbers apostichopus japonicus exhibits potent anticancer activity in A549 cancer cell line. Chin J Analyt Chemist. (2021) 49:37–42. 10.1016/j.cjac.2021.05.001

[80] Suganya AM Sanjivkumar M Chandran MN Palavesam A Immanuel G. Pharmacological importance of sulphated polysaccharide carrageenan from red seaweed kappaphycus alvarezii in comparison with commercial carrageenan. Biomed Pharmacother. (2016) 84:1300–12. 10.1016/j.biopha.2016.10.067 27810787

[81] Benard C Cultrone A Michel C Rosales C Segain J-P Lahaye M. Degraded carrageenan causing colitis in rats induces tnf secretion and Icam-1 upregulation in monocytes through Nf-κb activation. PLoS ONE. (2010) 5:e8666. 10.1371/journal.pone.0008666 20072622 PMC2800179

[82] Wei W Feng W Xin G Tingting N Zhanghe Z Haimin C. Enhanced effect of κ-Carrageenan on Tnbs-induced inflammation in mice. Int Immunopharmacol. (2016) 39:218–28. 10.1016/j.intimp.2016.07.031 27494685

[83] Wu W Zhen Z Niu T Zhu X Gao Y Yan J. κ-Carrageenan enhances lipopolysaccharide-induced interleukin-8 secretion by stimulating the Bcl10-Nf-κb pathway in Ht-29 cells and aggravates C. freundii-induced inflammation in mice. Mediat Inflamm. (2017) 2017:8634865. 10.1155/2017/8634865 28163398 PMC5253498

[84] Mi Y Chin YX Cao WX Chang YG Lim PE Xue CH. Native κ-Carrageenan induced-colitis is related to host intestinal microecology. Int J Biol Macromol. (2020) 147:284–94. 10.1016/j.ijbiomac.2020.01.072 31926226

[85] Sun Y Cui X Duan M Ai C Song S Chen X. In vitro fermentation of κ-carrageenan oligosaccharides by human gut microbiota and its inflammatory effect on ht29 cells. J Funct Foods. (2019) 59:80–91. 10.1016/j.jff.2019.05.036

[86] Hsu H-Y Hwang P-A. Clinical applications of fucoidan in translational medicine for adjuvant cancer therapy. Clin Transl Med. (2019) 8:1–18. 10.1186/s40169-019-0234-9 31041568 PMC6491526

[87] Dai Y-L Jiang Y-F Lu Y-A Kang M-C Jeon Y-J. Fucoidan from acid-processed hizikia fusiforme attenuates oxidative damage and regulate apoptosis. Int J Biol Macromol. (2020) 160:390–7. 10.1016/j.ijbiomac.2020.05.143 32446896

[88] Chollet L Saboural P Chauvierre C Villemin J-N Letourneur D Chaubet F. Fucoidans in nanomedicine. Mar Drugs. (2016) 14:145. 10.3390/md14080145 27483292 PMC4999906

[89] Takahashi H Kawaguchi M Kitamura K Narumiya S Kawamura M Tengan I. An exploratory study on the anti-inflammatory effects of fucoidan in relation to quality of life in advanced cancer patients. Integr Cancer Ther. (2018) 17:282–91. 10.1177/1534735417692097 28627320 PMC6041928

[90] Fitton JH Stringer DN Karpiniec SS. Therapies from fucoidan: an update. Mar Drugs. (2015) 13:5920–46. 10.3390/md13095920 26389927 PMC4584361

[91] Xue M Liang H Ji X Zhou Z Liu Y Sun T. Effects of fucoidan on gut flora and tumor prevention in 1, 2-dimethylhydrazine-induced colorectal carcinogenesis. J Nutr Biochem. (2020) 82:108396. 10.1016/j.jnutbio.2020.108396 32388163

[92] Kim I-H Nam T-J. Fucoidan downregulates insulin-like growth factor-I receptor levels in Ht-29 human colon cancer cells. Oncol Rep. (2018) 39:1516–22. 10.3892/or.2018.6193 29328495

[93] Pangestuti R Kim S-K. Biological activities and health benefit effects of natural pigments derived from marine algae. J Funct Foods. (2011) 3:255–66. 10.1016/j.jff.2011.07.001

[94] Osório C Machado S Peixoto J Bessada S Pimentel FBC Alves R. Pigments content (chlorophylls, fucoxanthin and phycobiliproteins) of different commercial dried algae. Separations. (2020) 7:33. 10.3390/separations7020033

[95] Pérez MJ Falqué E Domínguez H. Antimicrobial action of compounds from marine seaweed. Mar Drugs. (2016) 14:52. 10.3390/md14030052 27005637 PMC4820306

[96] Wang H-MD Li X-C Lee D-J Chang J-S. Potential biomedical applications of marine algae. Bioresour Technol. (2017) 244:1407–15. 10.1016/j.biortech.2017.05.198 28697977

[97] Martínez Andrade KA Lauritano C Romano G Ianora A. Marine microalgae with anti-cancer properties. Mar Drugs. (2018) 16:165. 10.3390/md16050165 29762545 PMC5983296

[98] Smyrniotopoulos V Vagias C Bruyère C Lamoral-Theys D Kiss R Roussis V. Structure and in vitro antitumor activity evaluation of brominated diterpenes from the red alga sphaerococcus coronopifolius. Bioorg Med Chem. (2010) 18:1321–30. 10.1016/j.bmc.2009.12.025 20045651

[99] Bandaranayake WM. The nature and role of pigments of marine invertebrates. Nat Prod Rep. (2006) 23:223–55. 10.1039/b307612c 16572229

[100] Manivasagan P Bharathiraja S Santha Moorthy M Mondal S Seo H Dae Lee K. Marine natural pigments as potential sources for therapeutic applications. Crit Rev Biotechnol. (2018) 38:745–61. 10.1080/07388551.2017.1398713 29124966

[101] Sathasivam R Ki J-S. A Review of the biological activities of microalgal carotenoids and their potential use in healthcare and cosmetic industries. Mar Drugs. (2018) 16:26. 10.3390/md16010026 29329235 PMC5793074

[102] Martin LJ. Fucoxanthin and its metabolite fucoxanthinol in cancer prevention and treatment. Mar Drugs. (2015) 13:4784–98. 10.3390/md13084784 26264004 PMC4557004

[103] Chuyen HV Eun J-B. Marine carotenoids: bioactivities and potential benefits to human health. Crit Rev Food Sci Nutr. (2017) 57:2600–10. 10.1080/10408398.2015.1063477 26565683

[104] Mondal A Bose S Banerjee S Patra JK Malik J Mandal SK. Marine cyanobacteria and microalgae metabolites—a rich source of potential anticancer drugs. Mar Drugs. (2020) 18:476. 10.3390/md18090476 32961827 PMC7551136

[105] Komba S Kotake-Nara E Tsuzuki W. Degradation of fucoxanthin to elucidate the relationship between the fucoxanthin molecular structure and its antiproliferative effect on Caco-2 cells. Mar Drugs. (2018) 16:275. 10.3390/md16080275 30082622 PMC6117710

[106] Nakazawa Y Sashima T Hosokawa M Miyashita K. Comparative evaluation of growth inhibitory effect of stereoisomers of fucoxanthin in human cancer cell lines. J Funct Foods. (2009) 1:88–97. 10.1016/j.jff.2008.09.015

[107] Terasaki M Mima M Kudoh S Endo T Maeda H Hamada J. Glycine and succinic acid are effective indicators of the suppression of epithelial-mesenchymal transition by fucoxanthinol in colorectal cancer stem-like cells. Oncol Rep. (2018) 40:414–24. 10.3892/or.2018.6398 29693702

[108] Tamura S Narita T Fujii G Miyamoto S Hamoya T Kurokawa Y. Inhibition of Nf-Kappab transcriptional activity enhances fucoxanthinol-induced apoptosis in colorectal cancer cells. Genes Environ. (2019) 41:1–8. 10.1186/s41021-018-0116-1 30693059 PMC6341523

[109] Takahashi K Hosokawa M Kasajima H Hatanaka K Kudo K Shimoyama N. Anticancer effects of fucoxanthin and fucoxanthinol on colorectal cancer cell lines and colorectal cancer tissues. Oncol Lett. (2015) 10:1463–7. 10.3892/ol.2015.3380 26622691 PMC4533304

[110] Terasaki M Hamoya T Kubota A Kojima H Tanaka T Maeda H. Fucoxanthin prevents colorectal cancer development in dextran sodium sulfate-treated Apcmin/+ Mice. Anticancer Res. (2021) 41:1299–305. 10.21873/anticanres.14887 33788721

[111] Terasaki M Uehara O Ogasa S Sano T Kubota A Kojima H. Alteration of fecal microbiota by fucoxanthin results in prevention of colorectal cancer in Aom/Dss mice. Carcinogenesis. (2021) 42:210–9. 10.1093/carcin/bgaa100 32940665

[112] Terasaki M Ikuta M Kojima H Tanaka T Maeda H Miyashita K. Dietary fucoxanthin induces anoikis in colorectal adenocarcinoma by suppressing integrin signaling in a murine colorectal cancer model. J Clin Med. (2019) 9:90. 10.3390/jcm9010090 31905803 PMC7019251

[113] Terasaki M Murase W Kamakura Y Kawakami S Kubota A Kojima H. A biscuit containing fucoxanthin prevents colorectal carcinogenesis in mice. Nutr Cancer. (2022) 2022:1–11. 10.1080/01635581.2022.2086703 35695489

[114] Terasaki M Ono S Hashimoto S Kubota A Kojima H Ohta T. Suppression of Cc chemokine receptor 1 is a key regulation for colon cancer chemoprevention in Aom/Dss mice by fucoxanthin. J Nutr Biochem. (2022) 99:108871. 10.1016/j.jnutbio.2021.108871 34571188

[115] Palozza P Torelli C Boninsegna A Simone R Catalano A Mele MC. Growth-inhibitory effects of the astaxanthin-rich alga haematococcus pluvialis in human colon cancer cells. Cancer Lett. (2009) 283:108–17. 10.1016/j.canlet.2009.03.031 19423215

[116] Nagendraprabhu P Sudhandiran G. Astaxanthin inhibits tumor invasion by decreasing extracellular matrix production and induces apoptosis in experimental rat colon carcinogenesis by modulating the expressions of Erk-2, Nfκb and Cox-2. Invest New Drugs. (2011) 29:207–24. 10.1007/s10637-009-9342-5 19876598

[117] Kochi T Shimizu M Sumi T Kubota M Shirakami Y Tanaka T. Inhibitory effects of astaxanthin on azoxymethane-induced colonic preneoplastic lesions in C57/Bl/Ksj-Db/Dbmice. BMC Gastroenterol. (2014) 14:1–10. 10.1186/s12876-014-0212-z 25515685 PMC4273491

[118] Mišurcová L AmbroŽová J Samek D. Seaweed lipids as nutraceuticals. Adv Food Nutr Res. (2011) 64:339–55. 10.1016/B978-0-12-387669-0.00027-2 22054960

[119] Zárate R el Jaber-Vazdekis N Tejera N Pérez JA Rodríguez C. Significance of long chain polyunsaturated fatty acids in human health. Clin Translat Med. (2017) 6:1–19. 10.1186/s40169-017-0153-6 28752333 PMC5532176

[120] Zhang J Zhang L Ye X Chen L Zhang L Gao Y. Characteristics of fatty acid distribution is associated with colorectal cancer prognosis. Prostaglandins Leukot Essent Fatty Acids. (2013) 88:355–60. 10.1016/j.plefa.2013.02.005 23465412

[121] Zhang L-j Chen B Zhang J-j Li J Yang Q Zhong Q-s. Serum polyunsaturated fatty acid metabolites as useful tool for screening potential biomarker of colorectal cancer. Prostaglandins Leukot Essent Fatty Acids. (2017) 120:25–31. 10.1016/j.plefa.2017.04.003 28515019

[122] Rajapakse N Kim S-K. Nutritional and digestive health benefits of seaweed. Adv Food Nutr Res. (2011) 64:17–28. 10.1016/B978-0-12-387669-0.00002-8 22054935

[123] Cockbain A Toogood G Hull MA. Omega-3 polyunsaturated fatty acids for the treatment and prevention of colorectal cancer. Gut. (2012) 61:135–49. 10.1136/gut.2010.233718 21490374

[124] Granci V Cai F Lecumberri E Clerc A Dupertuis YM Pichard C. Colon cancer cell chemosensitisation by fish oil emulsion involves apoptotic mitochondria pathway. Br J Nutr. (2013) 109:1188–95. 10.1017/S000711451200308X 22874769

[125] Zhang C Yu H Ni X Shen S Das UN. Growth inhibitory effect of polyunsaturated fatty acids (Pufas) on colon cancer cells via their growth inhibitory metabolites and fatty acid composition changes. PLoS ONE. (2015) 10:e0123256. 10.1371/journal.pone.0123256 25886460 PMC4401647

[126] Wang W Yang J Nimiya Y Lee KSS Sanidad K Qi W. Ω-3 Polyunsaturated fatty acids and their cytochrome P450-derived metabolites suppress colorectal tumor development in mice. J Nutr Biochem. (2017) 48:29–35. 10.1016/j.jnutbio.2017.06.006 28672272 PMC6278818

[127] Huang Q Wen J Chen G Ge M Gao Y Ye X. Omega-3 polyunsaturated fatty acids inhibited tumor growth via preventing the decrease of genomic dna methylation in colorectal cancer rats. Nutr Cancer. (2016) 68:113–9. 10.1080/01635581.2016.1115526 26771229

[128] Hawcroft G Volpato M Marston G Ingram N Perry SL Cockbain AJ. The omega-3 polyunsaturated fatty acid eicosapentaenoic acid inhibits mouse mc-26 colorectal cancer cell liver metastasis via inhibition of Pge2-dependent cell motility. Br J Pharmacol. (2012) 166:1724–37. 10.1111/j.1476-5381.2012.01882.x 22300262 PMC3419914

[129] West NJ Clark SK Phillips RK Hutchinson JM Leicester RJ Belluzzi A. Eicosapentaenoic acid reduces rectal polyp number and size in familial adenomatous polyposis. Gut. (2010) 59:918–25. 10.1136/gut.2009.200642 20348368

[130] Cockbain AJ Volpato M Race AD Munarini A Fazio C Belluzzi A. Anticolorectal cancer activity of the omega-3 polyunsaturated fatty acid eicosapentaenoic acid. Gut. (2014) 63:1760–8. 10.1136/gutjnl-2013-306445 24470281

[131] Piazzi G D'Argenio G Prossomariti A Lembo V Mazzone G Candela M. Eicosapentaenoic acid free fatty acid prevents and suppresses colonic neoplasia in colitis-associated colorectal cancer acting on notch signaling and gut microbiota. Int J Cancer. (2014) 135:2004–13. 10.1002/ijc.28853 24676631

[132] D'Eliseo D Velotti F. Omega-3 fatty acids and cancer cell cytotoxicity: implications for multi-targeted cancer therapy. J Clin Med. (2016) 5:15. 10.3390/jcm5020015 26821053 PMC4773771

[133] Calviello G Di Nicuolo F Gragnoli S Piccioni E Serini S Maggiano N. N-3 Pufas reduce vegf expression in human colon cancer cells modulating the Cox-2/Pge 2 induced Erk-1 and-2 and Hif-1α induction pathway. Carcinogenesis. (2004) 25:2303–10. 10.1093/carcin/bgh265 15358633

[134] Giros A Grzybowski M Sohn VR Pons E Fernandez-Morales J Xicola RM. Regulation of colorectal cancer cell apoptosis by the n-3 polyunsaturated fatty acids docosahexaenoic and eicosapentaenoic. Cancer Prev Res. (2009) 2:732–42. 10.1158/1940-6207.CAPR-08-0197 19638488 PMC3793343

[135] Moradi Sarabi M Zahedi SA Pajouhi N Khosravi P Bagheri S Ahmadvand H. The effects of dietary polyunsaturated fatty acids on Mir-126 promoter DNA methylation status and vegf protein expression in the colorectal cancer cells. Genes Nutr. (2018) 13:1–9. 10.1186/s12263-018-0623-5 30598703 PMC6299631

[136] Rodríguez-González I Díaz-Reinoso B Domínguez H. Intensification strategies for the extraction of polyunsaturated fatty acids and other lipophilic fractions from seaweeds. Food Bioproc Technol. (2022) 15:1–20. 10.1007/s11947-021-02757-1

[137] Xu Y Qian SY. Anti-cancer activities of Ω-6 polyunsaturated fatty acids. Biomed J. (2014) 37:112-9. 10.4103/2319-4170.131378 24923568 PMC4166599

[138] Jones R Adel-Alvarez L-A Alvarez OR Broaddus R Das S. Arachidonic acid and colorectal carcinogenesis. Mol Cell Biochem. (2003) 253:141–9. 10.1023/A:1026060426569 14619964

[139] Lu X-f He G-q Yu H-n Ma Q Shen S-r Das UN. Colorectal cancer cell growth inhibition by linoleic acid is related to fatty acid composition changes. J Zhejiang Univ Sci B. (2010) 11:923–30. 10.1631/jzus.B1000125 21121070 PMC2997400

[140] Ohmori H Luo Y Fujii K Sasahira T Shimomoto T Denda A. Dietary linoleic acid and glucose enhances azoxymethane-induced colon cancer and metastases via the expression of high-mobility group box 1. Pathobiology. (2010) 77:210–7. 10.1159/000296305 20616616

[141] Pal A Kamthania MC Kumar A. Bioactive compounds and properties of seaweeds—a review. Open Access Library J. (2014) 1:1–17. 10.4236/oalib.1100752

[142] Sohn S-I Rathinapriya P Balaji S Jaya Balan D Swetha TK Durgadevi R. Phytosterols in seaweeds: an overview on biosynthesis to biomedical applications. Int J Mol Sci. (2021) 22:12691. 10.3390/ijms222312691 34884496 PMC8657749

[143] Jiang L Zhao X Xu J Li C Yu Y Wang W. The protective effect of dietary phytosterols on cancer risk: a systematic meta-analysis. J Oncol. (2019) 2019:7479518. 10.1155/2019/7479518 31341477 PMC6612402

[144] Sánchez-Machado D López-Hernández J Paseiro-Losada P López-Cervantes J. An Hplc method for the quantification of sterols in edible seaweeds. Biomed Chromatogr. (2004) 18:183–90. 10.1002/bmc.316 15103705

[145] Wang Z Zhan Y Xu J Wang Y Sun M Chen J. β-Sitosterol reverses multidrug resistance via bcrp suppression by inhibiting the P53–Mdm2 interaction in colorectal cancer. J Agric Food Chem. (2020) 68:3850–8. 10.1021/acs.jafc.0c00107 32167760

[146] Shathviha PC Ezhilarasan D Rajeshkumar S Selvaraj J. β-Sitosterol mediated silver nanoparticles induce cytotoxicity in human colon cancer Ht-29 cells. Avicenna J Med Biotechnol. (2021) 13:42-6. 10.18502/ajmb.v13i1.4577 33680372 PMC7903430

[147] Arul AB Al Numair K Al Saif M Savarimuthu I. Effect of Dietary β-Sitosterol on Fecal Bacterial and Colonic Biotransformation Enzymes in 1, 2-Dimethylhydrazine–Induced Colon Carcinogenesis. Turkish J Med Sci. (2012) 42:1307–13. 10.3906/sag-1106-16

[148] Abdul QA Choi RJ Jung HA Choi JS. Health benefit of fucosterol from marine algae: a review. J Sci Food Agric. (2016) 96:1856–66. 10.1002/jsfa.7489 26455344

[149] Lee D-G Park J-H Yoo K-H Chung I-S Lee Y-H Lee J-K. 24-Ethylcholesta-4, 24 (28)-Dien-3, 6-Dione from osmanthus fragrans var. aurantiacus flowers inhibits the growth of human colon cancer cell line, Hct-116. J Korean Soc Appl Biol Chemist. (2011) 54:206–10. 10.3839/jksabc.2011.034

[150] Leal MC Munro MH Blunt JW Puga J Jesus B Calado R. Biogeography and biodiscovery hotspots of macroalgal marine natural products. Nat Prod Rep. (2013) 30:1380–90. 10.1039/c3np70057g 23982267

[151] Rabi T Bishayee A. Terpenoids and breast cancer chemoprevention. Breast Cancer Res Treat. (2009) 115:223–39. 10.1007/s10549-008-0118-y 18636327

[152] Rocha DH Seca AM Pinto DC. Seaweed secondary metabolites in vitro and in vivo anticancer activity. Mar Drugs. (2018) 16:410. 10.3390/md16110410 30373208 PMC6266495

[153] Huang M Lu J-J Huang M-Q Bao J-L Chen X-P Wang Y-T. Terpenoids: natural products for cancer therapy. Expert Opin Investig Drugs. (2012) 21:1801–18. 10.1517/13543784.2012.727395 23092199

[154] Andrianasolo EH France D Cornell-Kennon S Gerwick WH. DNA Methyl transferase inhibiting halogenated monoterpenes from the madagascar red marine alga portieria h ornemannii. J Nat Prod. (2006) 69:576–9. 10.1021/np0503956 16643029

[155] Cotas J Leandro A Monteiro P Pacheco D Figueirinha A Gonçalves AM. Seaweed phenolics: from extraction to applications. Mar Drugs. (2020) 18:384. 10.3390/md18080384 32722220 PMC7460554

[156] Isaza Martínez JH Torres Castañeda HG. Preparation and chromatographic analysis of phlorotannins. J Chromatogr Sci. (2013) 51:825–38. 10.1093/chromsci/bmt045 23592824

[157] Catarino MD Silva A Cruz MT Mateus N Silva AM Cardoso SM. Phlorotannins from fucus vesiculosus: modulation of inflammatory response by blocking Nf-κb signaling pathway. Int J Mol Sci. (2020) 21:6897. 10.3390/ijms21186897 32962250 PMC7554702

[158] Kang M-H Kim I-H Nam T-J. Phloroglucinol induces apoptosis through the regulation of insulin-like growth factor 1 receptor signaling pathways in human colon cancer Ht-29 cells. Int J Oncol. (2014) 45:1036–42. 10.3892/ijo.2014.2521 24970012 PMC4121399

[159] Yi L Wang Q Luo H Lei D Tang Z Lei S. Inhibitory effects of polyphenols-rich components from three edible seaweeds on inflammation and colon cancer in vitro. Front Nutr. (2022) 9:856273. 10.3389/fnut.2022.856273 35634377 PMC9136665

[160] Eo HJ Kwon T-H Park GH Song HM Lee S-J Park N-H. In vitro anticancer activity of phlorofucofuroeckol a via upregulation of activating transcription factor 3 against human colorectal cancer cells. Mar Drugs. (2016) 14:69. 10.3390/md14040069 27043582 PMC4849073

[161] Karthik R Manigandan V Sheeba R Saravanan R Rajesh PR. Structural characterization and comparative biomedical properties of phloroglucinol from indian brown seaweeds. J Appl Phycol. (2016) 28:3561–73. 10.1007/s10811-016-0851-2

[162] Pereira L. A review of the nutrient composition of selected edible seaweeds. Seaweed Ecol Nutr Composit Med Use. (2011) 7:15–47.

[163] Song M Nishihara R Wang M Chan AT Qian ZR Inamura K. Plasma 25-Hydroxyvitamin D and colorectal cancer risk according to tumour immunity status. Gut. (2016) 65:296–304. 10.1136/gutjnl-2014-308852 25591978 PMC4503524

[164] Ng K Meyerhardt JA Wu K Feskanich D Hollis BW Giovannucci EL. Circulating 25-Hydroxyvitamin D levels and survival in patients with colorectal cancer. J Clin Oncol. (2008) 26:2984–91. 10.1200/JCO.2007.15.1027 18565885

[165] Gorham ED Garland CF Garland FC Grant WB Mohr SB Lipkin M. Vitamin D and prevention of colorectal cancer. J Steroid Biochem Mol Biol. (2005) 97:179–94. 10.1016/j.jsbmb.2005.06.018 16236494

[166] Newmark HL Yang K Kurihara N Fan K Augenlicht LH Lipkin M. Western-style diet-induced colonic tumors and their modulation by calcium and vitamin D in C57bl/6 mice: a preclinical model for human sporadic colon cancer. Carcinogenesis. (2009) 30:88–92. 10.1093/carcin/bgn229 19017685 PMC2722141

[167] Rawson JB Sun Z Dicks E Daftary D Parfrey PS Green RC. Vitamin D intake is negatively associated with promoter methylation of the wnt antagonist gene Dkk1 in a large group of colorectal cancer patients. Nutr Cancer. (2012) 64:919–28. 10.1080/01635581.2012.711418 22966878 PMC4323165

[168] Ferrer-Mayorga G Larriba MJ Crespo P Muñoz A. Mechanisms of action of vitamin D in colon cancer. J Steroid Biochem Mol Biol. (2019) 185:1–6. 10.1016/j.jsbmb.2018.07.002 29981368

[169] Yun J Mullarky E Lu C Bosch KN Kavalier A Rivera K. Vitamin C selectively kills kras and braf mutant colorectal cancer cells by targeting gapdh. Science. (2015) 350:1391–6. 10.1126/science.aaa5004 26541605 PMC4778961

[170] Matos GS Pereira SG Genisheva ZA Gomes AM Teixeira JA Rocha CM. Advances in extraction methods to recover added-value compounds from seaweeds: sustainability and functionality. Foods. (2021) 10:516. 10.3390/foods10030516 33801287 PMC7998159

[171] Michalak I Tiwari R Dhawan M Alagawany M Farag MR Sharun K. Antioxidant effects of seaweeds and their active compounds on animal health and production–a review. Vet Quarter. (2022) 42:48–67. 10.1080/01652176.2022.2061744 35363108 PMC9004519

[172] Venturelli S Leischner C Helling T Renner O Burkard M Marongiu L. Minerals and cancer: overview of the possible diagnostic value. Cancers. (2022) 14:1256. 10.3390/cancers14051256 35267564 PMC8909570

[173] Lee J Shin A Choi J-Y Kang D Lee J-K. Adherence to the Recommended Intake of Calcium and Colorectal Cancer Risk in the Hexa Study. Cancer Res Treat. (2021) 53:140. 10.4143/crt.2020.480 32854492 PMC7812010

[174] Ali MS Hussein RM Kandeil MA. The pro-oxidant, apoptotic and anti-angiogenic effects of selenium supplementation on colorectal tumors induced by 1, 2-dimethylhydrazine in Balb/C mice. Rep Biochem Mol Biol. (2019) 8:216–26. 32274393 PMC7103074

